# PD-L1 Expression in Circulating Tumor Cells Increases during Radio(chemo)therapy and Indicates Poor Prognosis in Non-small Cell Lung Cancer

**DOI:** 10.1038/s41598-018-36096-7

**Published:** 2019-01-24

**Authors:** Yang Wang, Tae Hyun Kim, Shamileh Fouladdel, Zhuo Zhang, Payal Soni, Angel Qin, Lili Zhao, Ebrahim Azizi, Theodore S. Lawrence, Nithya Ramnath, Kyle C. Cuneo, Sunitha Nagrath

**Affiliations:** 1 Department of Chemical Engineering, University of Michigan, Ann Arbor, MI USA; 2 Biointerfaces Institute, University of Michigan, Ann Arbor, MI USA; 3 Department of Electrical Engineering and Computer Science, University of Michigan, Ann Arbor, MI USA; 4 Department of Internal Medicine, University of Michigan Comprehensive Cancer Center, Ann Arbor, MI USA; 5 Department of Radiation Oncology, University of Michigan Medical School, Ann Arbor, MI USA; 6 Department of Biostatistics, University of Michigan, Ann Arbor, MI USA; 7 Veterans Administration Ann Arbor Healthcare System, Ann Arbor, MI 48105 USA; 8 Translational Oncology Program, University of Michigan Health System, Ann Arbor, MI USA

## Abstract

Preclinical studies demonstrated that radiation up-regulates PD-L1 expression in tumor cells, providing a rationale for combining PD-1/PD-L1 inhibitors with radiation. However this has not been validated in patients with non-small cell lung cancer due to the difficulty to obtain serial biopsies. Measuring PD-L1 expression in circulating tumor cells (CTCs), may allow real-time monitoring of immune activation in tumor. In this study, whole blood from non-metastatic NSCLC patients was collected before, during, and after radiation or chemoradiation using a microfluidic chip. PD-L1 expression in CTCs was assessed by immunofluorescence and qPCR and monitored through the course of treatment. Overall, PD-L1(+) CTCs were detected in 25 out of 38 samples (69.4%) with an average of 4.5 cells/ml. After initiation of radiation therapy, the proportion of PD-L1(+) CTCs increased significantly (median 0.7% vs. 24.7%, P < 0.01), indicating up-regulation of PD-L1 in tumor cells in response to radiation. In addition, patients positive for PD-L1 (≥5% of CTCs positive for PD-L1) at baseline had shorter PFS. Gene expression analysis revealed that higher levels of PD-L1 were associated with poor prognosis. Therefore, CTCs can be used to monitor dynamic changes of PD-L1 during radiation therapy which is potentially prognostic of response to treatment.

## Introduction

Lung cancer is the leading cause of cancer-related death in the U.S. and worldwide, with non–small cell lung cancer (NSCLC) accounting for over 80% of those cases^1,2^. Non-metastatic NSCLC patients who are medically inoperable or unresectable are generally offered radiotherapy with or without concurrent chemotherapy which yields 5-year overall survival rates ranging from 10–35%^3–5^. Better treatment options are greatly needed for these patients.

Recent developments in immunotherapy have started a new era in the treatment of NSCLC. Programmed death 1 (PD-1) receptor and its ligand (PD-L1) are key checkpoint proteins for regulating the antitumor immune responses^6^. The binding of PD-L1 to PD-1 can inhibit T cell function and proliferation and result in immune tolerance. As PD-L1 expression has been found in various tumors including NSCLC, the blockage of PD-1/PD-L1 has emerged as a new therapeutic approach that can restore the antitumor immunity^7^.

Recent clinical trials using PD-1/PD-L1 inhibitors have shown improved overall survival in NSCLC patients^8–10^. Based on data from the recent phase 3 trial, the PD-1 inhibitor pembrolizumab was approved by the U.S. Food and Drug Administration (FDA) for the first-line treatment of metastatic NSCLC whose tumors have 50 percent or more PD-L1 expression with no EGFR or ALK genomic tumor aberration^11^. To further improve the response rate and duration and to extend the benefit to additional patients, the idea of combining anti–PD-1/PD-L1 therapies with radiation or chemoradiation has been proposed and tested in clinical trials in non-metastatic NSCLC patients^12–14^. Growing evidence demonstrates that radiation can elicit an adaptive immune response, but the immunogenic effect of radiation could be undermined by the upregulation of PD-L1 in tumor microenvironment^15^. This provides the primary rationale for combining PD-1/PD-L1 inhibitors with radiation^16,17^. However, the upregulation of PD-L1 expression during radiation has not been validated among NSCLC patients because it is challenging to obtain serial biopsies during a course of therapy to monitor the PD-L1 expression in intrathoracic tumors.

The isolation of circulating tumor cells (CTCs) from peripheral blood provides a minimally invasive method to repeatedly sample tumor cells from the patient and monitor PD-L1 expression on tumor cells over time. The potential of CTCs as a prognostic and surrogate biomarker for NSCLC has been investigated using the FDA approved CellSearch System^18–21^. However, due to the relativity low yield of this assay, the CellSearch system has been reported to underestimate the number of CTCs and has a limited ability to detect CTCs in non-metastatic NSCLC patients, which largely limits its clinical utility in this patient population^22^.

Microfluidic-based CTC isolation technologies have emerged as an approach to capture CTCs with high sensitivity and have demonstrated the capacity to characterize the molecular traits of tumors, such as EGFR mutations^18,23–26^. Previously we developed a nanomaterial-based microfluidic platform for CTC isolation, the graphene oxide (GO) Chip, which consists of a microfluidic chamber and a substrate coated with GO nanosheets where the antibodies are tethered^27^. This technology takes advantage of the increased surface area afforded by GO to achieve higher antibody coating density, and thus improved sensitivity for CTC capture.

**Figure 1:**
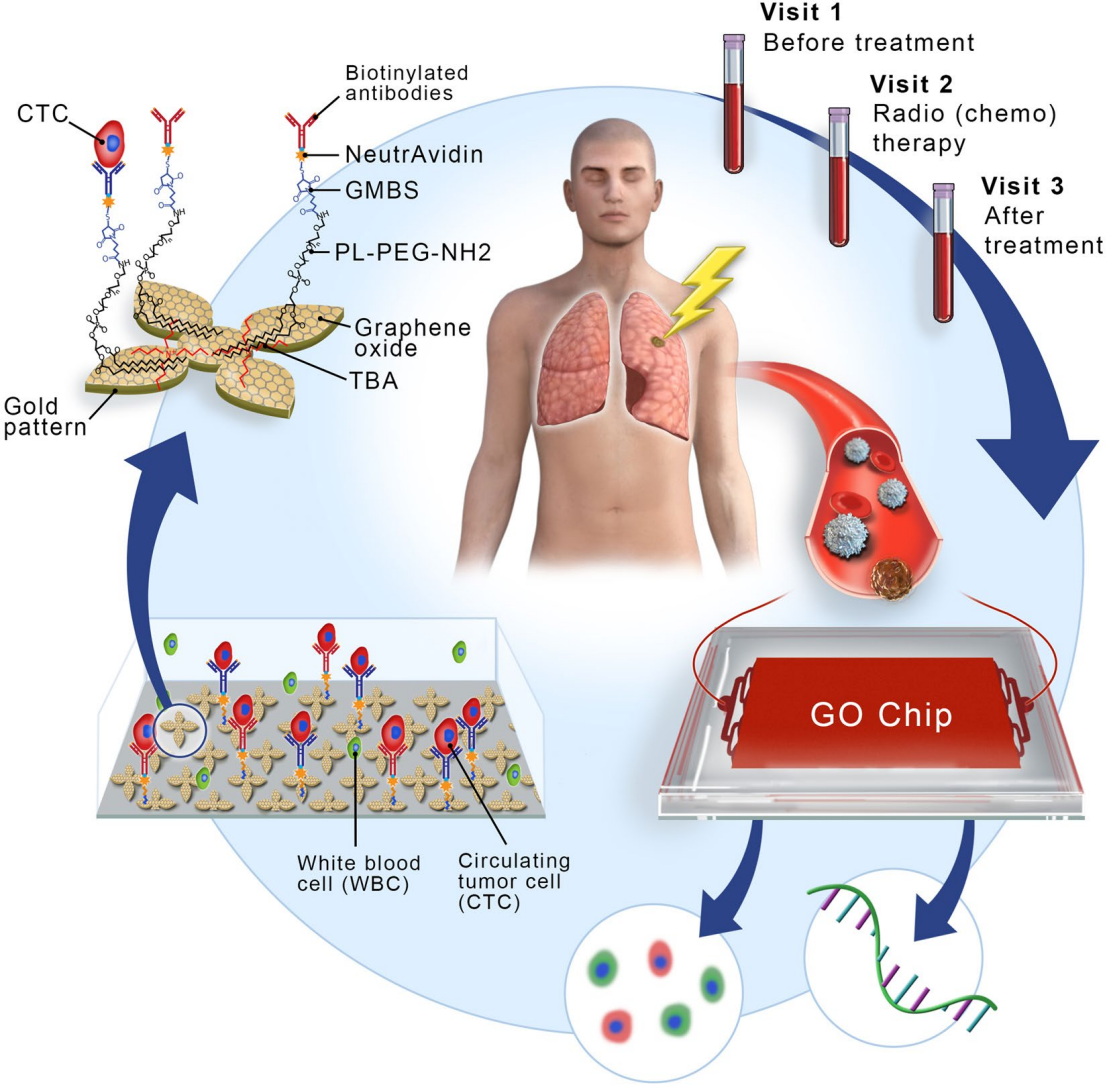
An overview of this study, with sample collection and circulating tumor cell (CTC) isolation before treatment (visit 1), during treatment (visit 2), and months after treatment (visit 3). The GO chip configuration and work mechanism is also shown by the schematic representations of CTC isolation within the microfluidic chamber and of antibody conjugation chemistry.

In this study, to investigate whether radiation therapy can increase PD-L1 expression in CTCs, we monitored the dynamic changes of PD-L1 expression in CTCs via the GO chip in 13 non-metastatic NSCLC patients who received radiation alone or with concurrent chemotherapy (Fig. 1). Furthermore, we evaluated whether PD-L1 (+) CTC counts and PD-L1 mRNA expression level correlates with patient outcomes.

## Results

### Isolation of lung cancer cells from model blood samples

**Figure 2:**
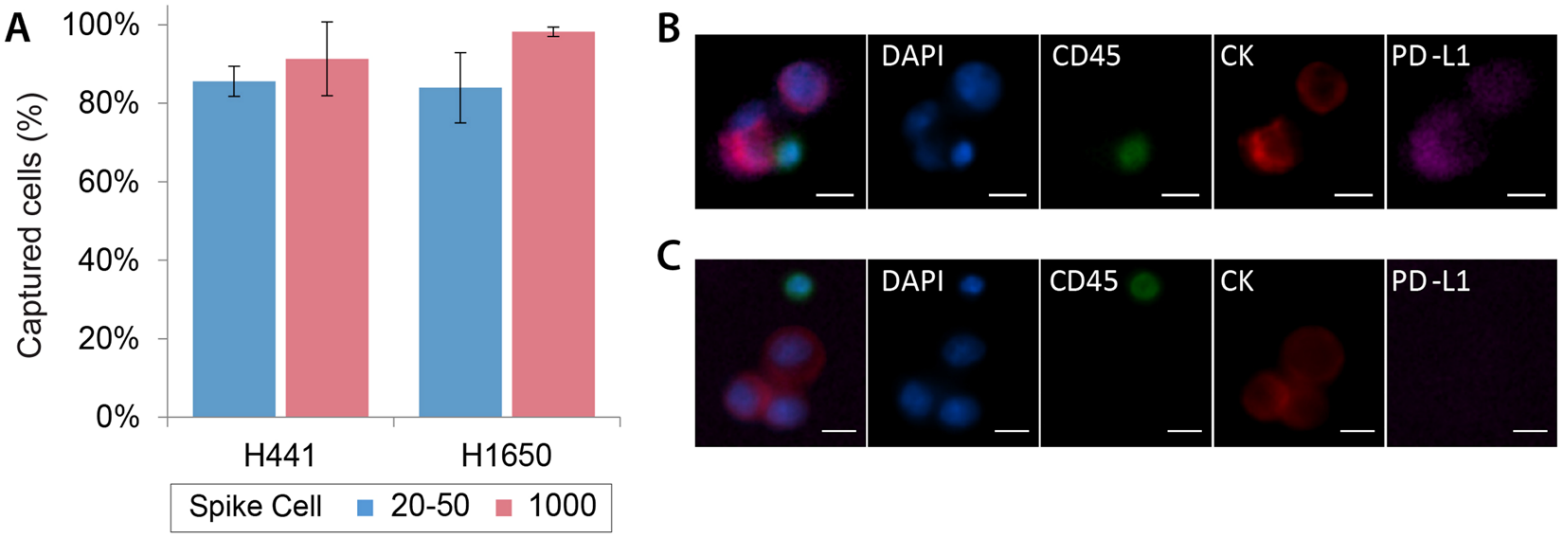
(**A**) Capture efficiency of lung cancer cell lines H441 (n = 3) and H1650 (n = 3). (**B**) Representative images of immunofluorescence staining of H441 lung cancer cells along with WBCs captured on the chip. (**C**) Representative images of immunofluorescence staining of H1650 lung cancer cells along with WBCs captured on the chip.

To test the performance of the GO device for NSCLC CTC capture, varying number of lung cancer cell lines, H1650 and H441 cells were labeled with green cell tracker dye and spiked into 1 ml of whole blood obtained from healthy donors. All samples were processed through the GO chip at a flow rate of 1 ml/hr. The total capture efficiency of H441 and H1650 cell lines using 1000 cells spiked into blood was 91.4 ± 9.4% and 98.2 ± 1.2% respectively. Low number of cells (20–50 cells) were also tested to mimic the rare nature of CTCs which resulted in an overall capture efficiency of 85.6 ± 3.8% and 84.0 ± 8.9% for H441 and H1650 cells (Fig. 2A).

To test the specificity of the PD-L1 staining antibody, the two cell lines without the green tracker dye were captured on chip followed by immunofluorescent analysis. All cells were stained for anti-Pan cytokeratin (CK) (tumor marker), anti-CD45 (leukocyte marker), anti-PD-L1 and DAPI (nuclear stain) and respective secondary antibodies. White blood cells were identified as positive for DAPI and CD45, while cancer cells were identified as positive for DAPI and CK, but negative for CD45. PD-L1 positive cell line, H441, was positively stained for PD-L1 (Fig. 2B) while no PD-L1 expression was detected in PD-L1 negative cell line H1650 (Fig. 2C). The concentrations of all staining antibody were tested and optimized to avoid any cross-reaction.

### Isolation of CTCs in non-metastatic NSCLC patients undergoing radiation or chemoradiation

Demographics of the 13 patients with NSCLC enrolled in the study are shown in Table S1. We collected serial blood samples from 13 patients with non-metastatic NSCLC who received radiation alone (n = 5) or chemoradiation (n = 8). Serial blood samples from the patients enrolled in the study were collected at the following time points: before the initiation of radiation (visit 1), during radiation (visit 2), and at follow up, approximately one month after radiation (visit 3). For each sample, 1 ml of blood was processed through single GO chips in parallel to analyze protein or gene expression patterns of CTCs. Due to the different duration of treatment in visit 2, whole blood was sampled twice for patient 1–11 and the number of CTCs for this visit was averaged. The data of visit 2 for patient 12 was obtained from a single sampling. Also, for patient 13, the CTC enumeration data of visit 2 was excluded due to the inadequate sampling volume for analysis. The flowchart in Fig. 1 provides an overview of the recruitment process and the method of CTC isolation.

**Figure 3:**
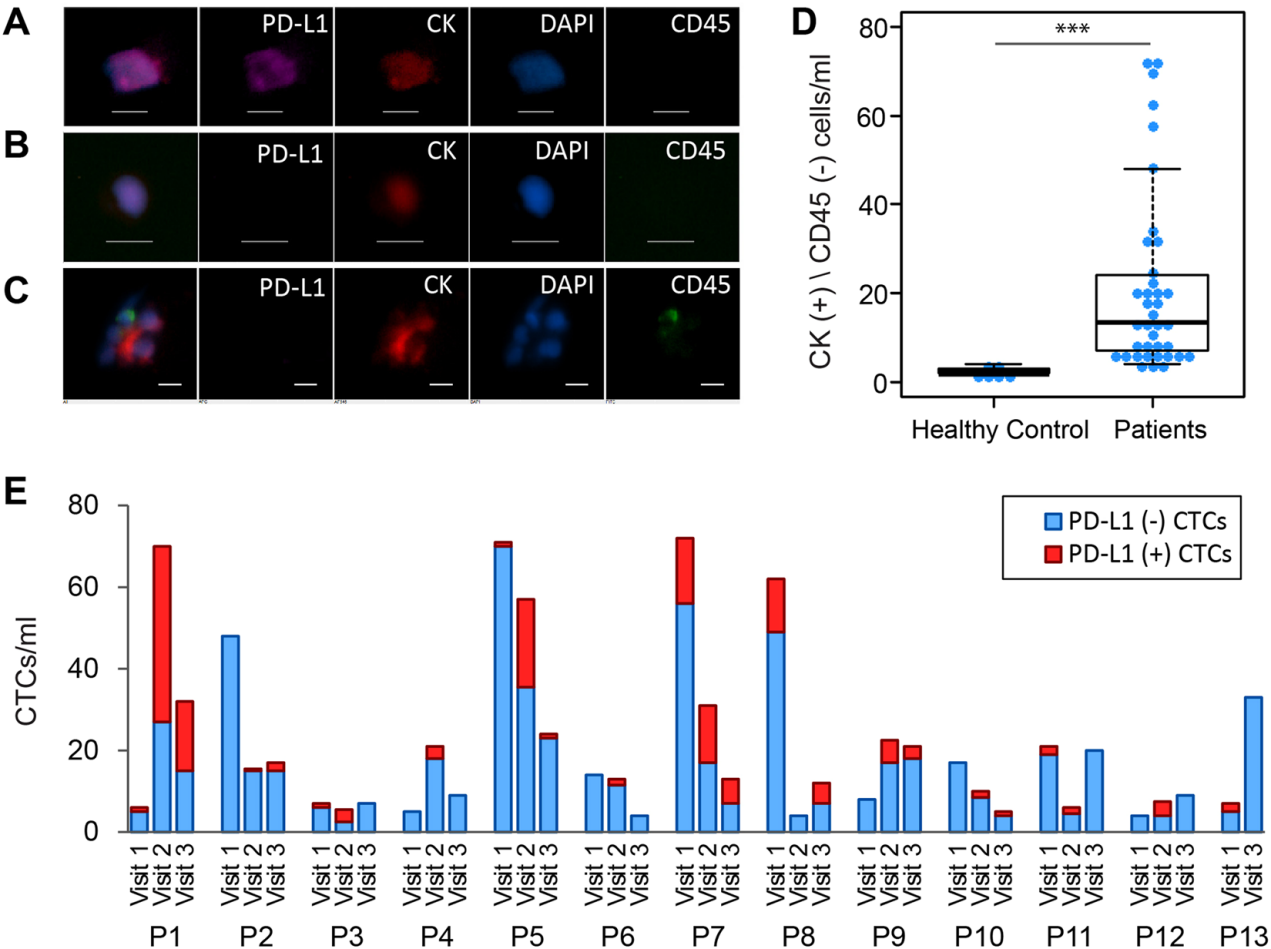
(**A**–**C**) Representative images of CTCs isolated from NSCLC patients stained by antibodies against cytokeratin (red), a leukocyte marker CD45 (green), and a nuclear stain (DAPI). (**A**) CK (+)/PD-L1 (+) CTC, (**B**) CK (+)/PD-L1 (−) CTC, (**C**) CTC cluster with WBC. Scale bar is 10 µm. (**D**) CTC enumeration from blood samples of healthy donors (n = 6) and from blood samples of NSCLC patients (n = 38), (***)P-value < 0.001. (**E**) Number of CTCs isolated by GO chip from blood samples from different visits of 13 NSCLC patients (P1 through P13). ‘P1’ stands for patient 1. Blue bar represents the number of CK (+)/PD-L1 (−) CTCs. Red bar represents the number of CK (+)/PD-L1 (+) CTCs.

After cells were captured on the GO chip, they were stained and imaged, and the number of CTCs was quantified as being CK+/CD45−/DAPI+ cells. Figure 3A,B show representative micrographs of PD-L1 (+) CTC and PD-L1 (−) CTC. While the majority of captured CTCs were observed in single cells, clusters of 2 to 3 CTCs were also detected in most patients (10/12) (Fig. 3C). Blood samples from healthy donors (n = 6) gave a counting of 2 to 3 CK(+)/CD45(−)/DAPI(+) cells/ml and thus a threshold of ≥4 CK(+)/CD45(−)/DAPI(+) cells/ml was used for CTC positivity in patient samples (Fig. 3D, Table S2). Overall, CTCs were detected in all 38 samples with an average of 21.3 CTCs/ml (range of 4–72 CTCs/ml) (Fig. 3E, Table S3). For the 8 patients receiving concurrent chemotherapy (patient 5 to 12), 6 (75%) showed a decrease in CTC number in visit 2 and visit 3 compared to visit 1 (Fig. 3E, Tables S1 and S3). For the 4 patients who received radiation therapy alone (patient 1 to 4), 2 patients had a 3 and 10 fold increase in CTC numbers during radiation (Fig. 3E, Tables S1 and S3).

### The changes of PD-L1 (+) CTC numbers in 12 NSCLC patients undergoing radiation or chemoradiation therapy

**Figure 4:**
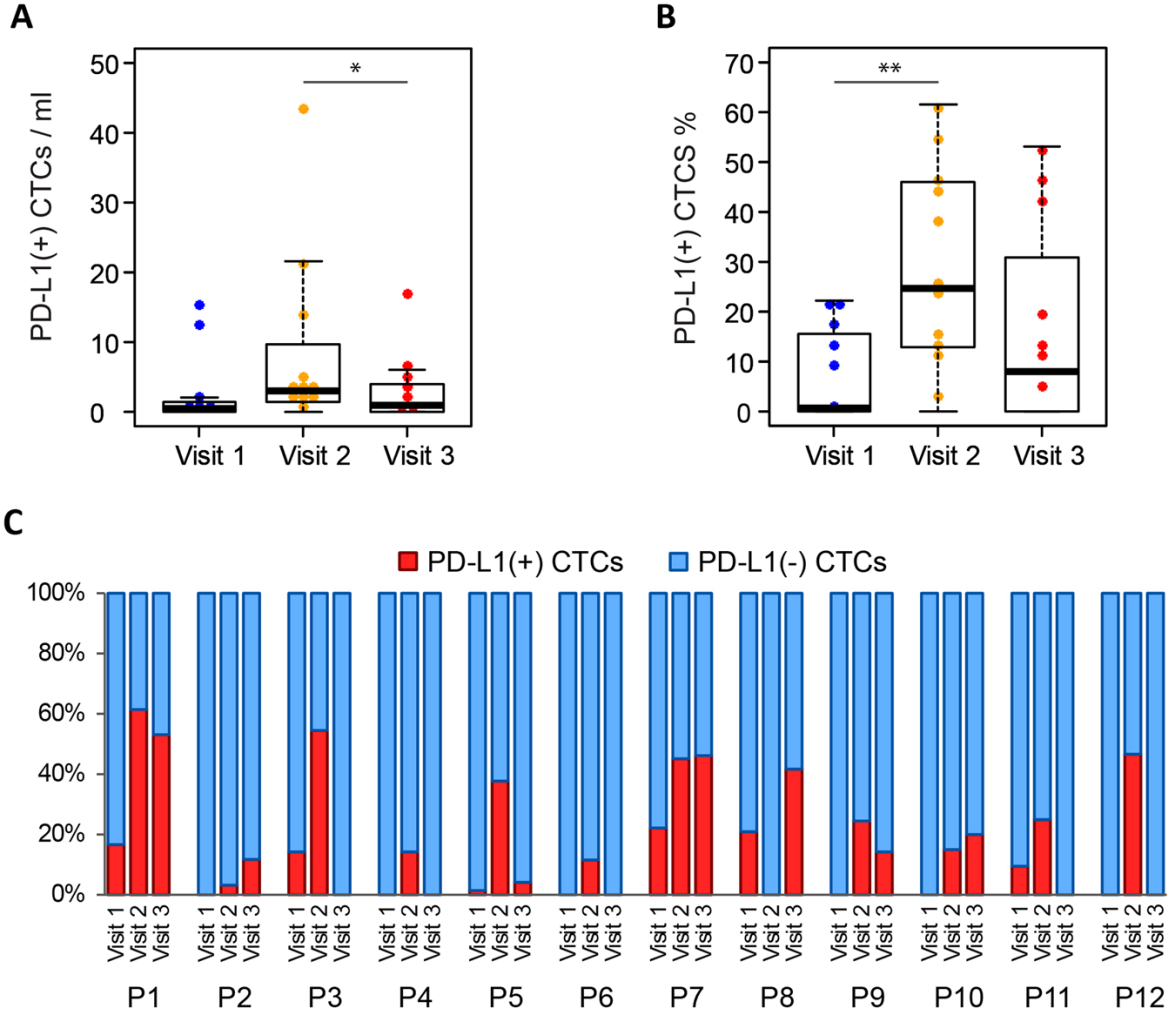
(**A**) The number of PD-L1 (+) CTCs in different visits (n = 12), (*)P-value < 0.05. (**B**) The proportion of PD-L1 (+) CTCs out of total CTCs in different visits (n = 12), (**)P-value < 0.01. (**C**) Dynamic changes of PD-L1 (+) CTC proportions at visit 1, visit 2, and visit 3 for 12 patients. ‘P1’ stands for patient 1. Red bar represents the percentage of PD-L1 (+) CTC number in total CTC number. Blue bar represents the percentage of PD-L1 (−) CTCs in total CTCs.

The changes of PD-L1 (+) CTC numbers during radiation therapy were analyzed from the 12 patients who had complete data on PD-L1 staining in CTCs for all time points (Table S3). PD-L1 (+) CTCs were detected in 24 (66.7%) out of 36 samples analyzed ranging from 0 to 43 PD-L1 (+) CTCs/ml (Fig. 3E, Table S3). The median number of PD-L1 (+) CTCs for visit 1 (pre-radiation), visit 2 (during radiation), and visit 3 (follow-up) were 0.5 CTCs/ml, 3 CTCs/ml, and 1 CTCs/ml respectively (Fig. 4A). All patients treated with radiation only (patient 1–4) have increased PD-L1 (+) CTC % during treatment (Fig. 4C). Similarly patients treated with concurrent carboplatin and paclitaxel (patient 5–12), 7 out of 8 patients showed increased PD-L1 (+) CTC % during treatment (Fig. 4C). Overall, the PD-L1 (+) CTC % is higher in visit 2 than that in visit 1 (median 0.7% vs 24.7%, p = 0.0068) (Fig. 4B, Table S4) suggesting that radiation therapy induces PD-L1 expression in CTCs.

### Prognostic significance of PD-L1 status in CTCs at baseline

**Figure 5:**
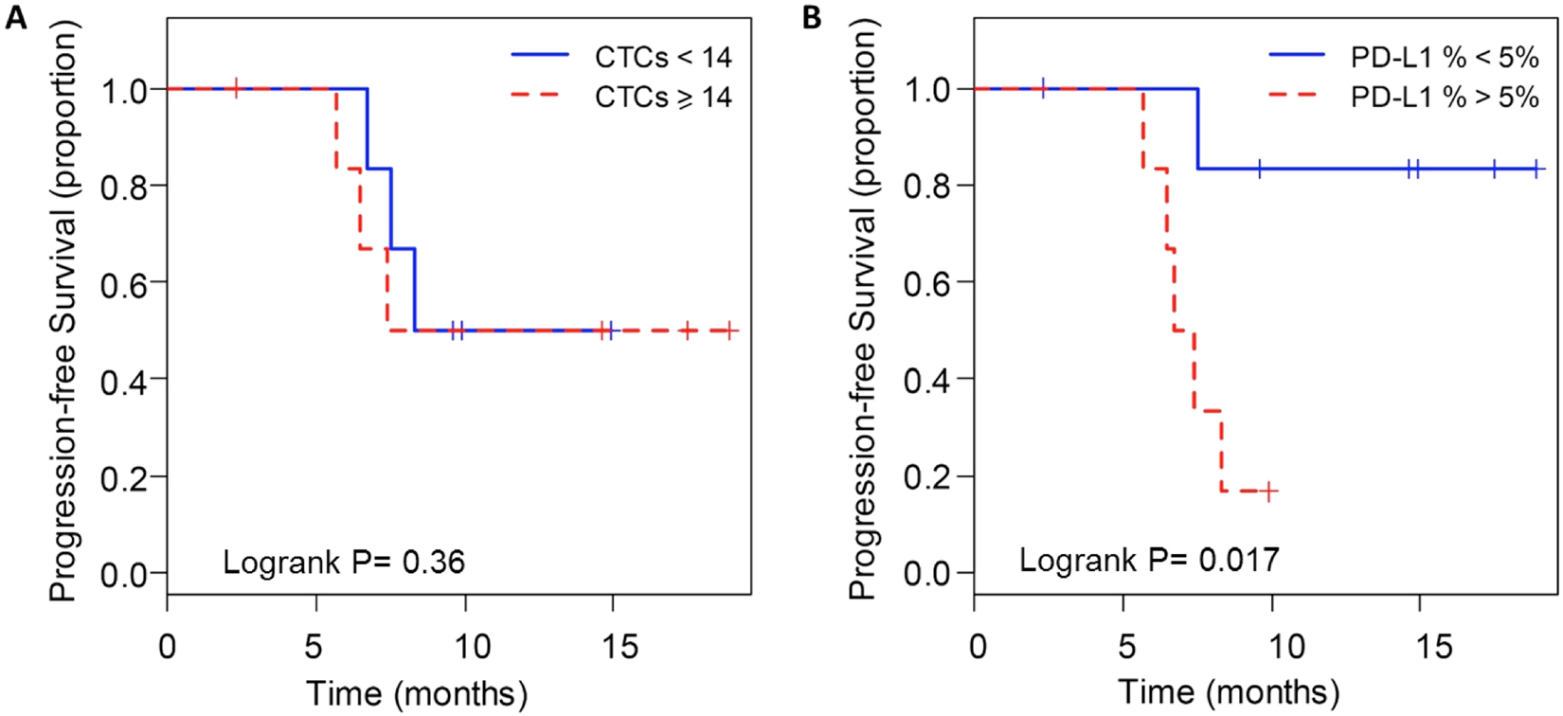
Kaplan–Meier life-table analysis of the PFS time in all patients. Grouping was done according to (**A**) CTC number more or less than 14/ml and (**B**) PD-L1 (+) CTC% more or less than 5%.

Progression-free survival (PFS) was analyzed according to the CTC number and the proportion of PD-L1 (+) CTCs at baseline (visit 1) for all patients who had adequate follow up data (Table S5). When the median number of CTCs (14 CTCs/ml) was chosen as the cut-off, there was no significant difference in PFS in patients with a high number of CTCs (≥14 CTCs/ml, median PFS 7.4 months) compared to those with a low number of CTCs (median PFS 9.6 months) (Fig. 5A) at baseline. To analyze the patient outcomes based on the proportion of PD-L1 (+) CTCs, a cutoff of 5% was applied which is commonly used to assess the PD-L1 positivity in tissue biopsy staining^28^. In result, PD-L1 positive patients had a shorter PFS compared to PD-L1 negative patients (median 7.1 months vs. median not reached: P = 0.017) (Fig. 5B). Notably, one of the patients who had a high PD-L1 (+) CTC counts at visit 2 and visit 3 was put on therapy with PD-1 inhibitor, pembrolizumab, after initial progression and has had stable disease for 7 months.

### Gene expression profiling of CTCs

**Figure 6:**
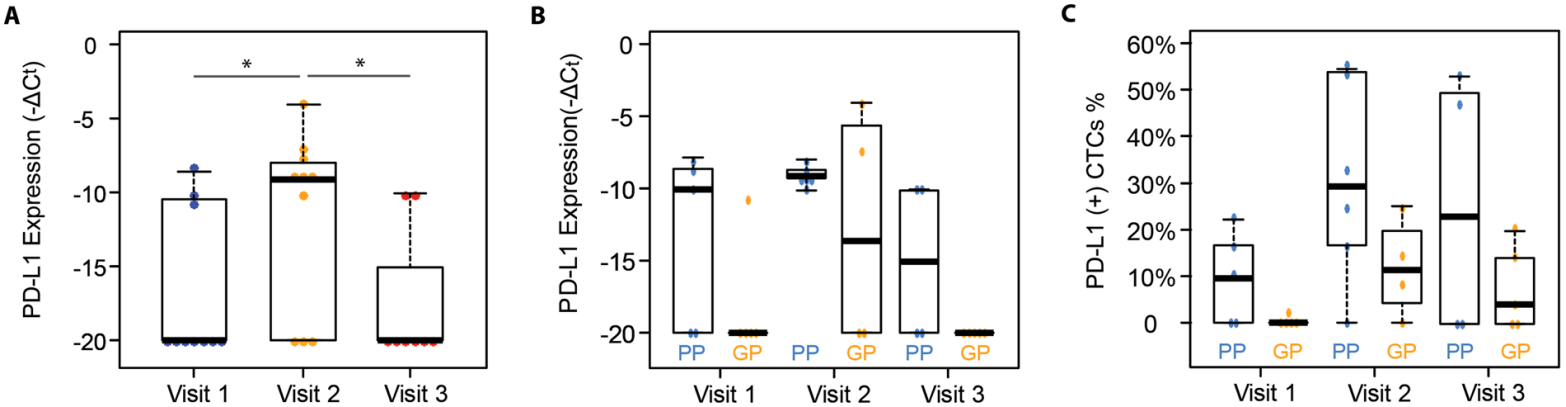
(**A**) mRNA level (−∆*C*_*t*_) of PD-L1 in visit 1 samples (n = 11), visit 2 samples (n = 11) and visit 3 samples (n = 9). (**B**) mRNA level (−∆*C*_*t*_) of PD-L1 (**B**) in PP samples (n = 15) versus GP samples (n = 14). (*)P-value < 0.05. (**C**) The proportion of PD-L1 (+) CTCs in total CTCs via immunostaining in PP samples (n = 17) versus GP samples (n = 18). PP stands for poor prognosis and GP stands for good prognosis.

We next investigated the molecular signature of CTCs from patients with lung cancer receiving radiation to study the association of these markers with clinical outcomes. Gene expression profiling of PD-L1 was carried out from the captured CTCs by RT-qPCR. After normalization with internal house-keeping genes (HKGs: GAPDH, ACTB, and UBB) using the ΔCt method, the PD-L1 mRNA expression levels at different visits were compared. The highest PD-L1 expression was seen in the visit 2 (during radiation) samples. 72.7% of these samples had detectable levels of PD-L1 mRNA expression. Whereas, 36.4% of visit 1 (pre-radiation) samples and 22.2% of visit 3 (follow-up) samples showed detectable PD-L1 gene expression (Fig. 6A). The expression levels of PD-L1 in visit 2 samples were significantly higher than those in visit 1 and visit 3 samples (P = 0.041 and P = 0.010, respectively), which is consistent with the results of PD-L1 (+) CTC counts. These data suggest that radiation induces PD-L1 expression in NSCLC CTCs and that this effect is transient.

To investigate the prognostic value of CTC gene expression, patients were classified into poor prognosis (PP) and good prognosis (GP) groups based off a cutoff at 9 month for PFS which was the median PFS for the entire cohort. The mRNA levels of PD-L1 is detected in 11 out of 15 samples from the PP group while the mRNA levels of PD-L1 is detected in 3 out of 14 samples from the GP group. The mRNA levels of PD-L1 were significantly higher in the samples from the PP group than those from the GP group (P = 0.026, Figs 6B and S1A). Similarly, from the results of immunofluorescent staining, PD-L1 (+) CTCs % in PP group is higher than that in the GP group (P = 0.042, Figs 6C and S1B). To account for the effect of contaminating WBCs in the isolated cells, we also analyzed the CD45 expression in the samples. No significant differences of the CD45 expression are observed between different visits (Fig. S2).

## Discussion

Preclinical studies have demonstrated that the combination of radiotherapy and PD-1/PD-L1 checkpoint blockade synergistically enhances antitumor immune activity and increases the treatment efficacy of either therapy alone^16,17^. A suggested mechanism is that the inflammatory response after radiation up-regulates the PD-L1 expression in tumor cells and surrounding cells, resulting in an immune-suppressive microenvironment^16,17^. These findings, however, have not been validated by tumor biopsy samples because it is challenging to obtain serial biopsies. CTC isolation provides a minimally invasive way to monitor PD-L1 expression in tumor cells over time. The feasibility of analyzing PD-L1 (+) CTCs has been demonstrated in breast cancer and NSCLC^29,30^. In a recent study, the persistence of PD-L1 (+) CTCs detected by CellSearch System was shown to be correlated with immunotherapy resistance in 14 metastatic NSCLC patients treated with PD-1 inhibitor Nivolumab, indicating that PD-L1 (+) CTCs may be a marker of immune escape^30^. However, the small number of PD-L1 (+) CTCs detected cannot reflect the dynamic changes of PD-L1 expression. In this study, we were able to detect sufficient number of CTCs via the GO chip at different time points to monitor the changes of PD-L1 expression in CTCs. Several features of the GO chip contribute to the improved sensitivity of CTC isolation compared to conventional methods: 1) graphene oxide increases the surface area on which the tumor specific capture antibodies were present, 2) microfluidic structure obtains optimized flow pattern for cell capture, 3) an antibody cocktail of anti-EpCAM, anti-EGFR and anti-CD133 enables the isolation of CTCs expressing variable levels of epithelial and mesenchymal markers. Furthermore, as the white blood cell contamination within the GO chip is very low, the captured CTCs maintained high purity and thus enabled downstream gene analysis^27^.

In this study, we demonstrate that for non-metastatic NSCLC patients, PD-L1 expression in CTCs is transiently upregulated during a course of radiation therapy. At baseline, 46.2% of samples showed PD-L1 staining in CTCs if a 5% threshold for positivity is used. This is in line with previous studies reporting that a range of 20% to 57.5% of solid biopsy samples from stage I-III NSCLC patients express PD-L1^31–33^. Radiation and chemoradiation are the standard therapies for this cohort of inoperable, non-metastatic NSCLC patients^34^. We analyzed serial blood samples from 4 patients treated with radiation and 8 patients treated with chemoradiation. All patients treated with radiation only have demonstrated increased PD-L1 expression during treatment. Among patients treated with concurrent carboplatin and paclitaxel, 7 out of 8 patients showed increased PD-L1 expression during treatment. Hence, we suggest that PD-L1 expression in CTC increases during radiation, with or without concurrent chemotherapy. Recently, Adams *et al*. showed that among lung cancer patients, PD-L1 expression in CTCs increased after the radiation started via immunostaining, which is consistent to our results^35^. Furthermore, we validated the upregulation of PD-L1 at the transcript level. The mRNA expression levels of PD-L1 in visit 2 (during treatment) samples were higher than that in visit 1 (pre-treatment) and visit 3 (>1 month after treatment), indicating that the upregulation of PD-L1 during radiation is transient. However, it is still unclear whether PD-L1 expression in CTCs is obtained in primary tumor before intravasation or could be acquired within the blood circulation. Future studies that compare the PD-L1 expression of tissue biopsies or surgical specimens with that of CTCs will be very helpful to investigate the mechanism of PD-L1 expression in CTCs and to understand the clinical utility of PD-L1 (+) CTCs.

Furthermore, we revealed that PD-L1 positivity in CTCs might serve as a prognostic marker among non-metastatic NSCLC patients receiving radiation therapy. PD-L1 expression is associated with worse PFS or OS in advanced lung cancer and gastrointestinal cancer^36,37^. Similarly, in our study for non-metastatic lung cancer, PD-L1 positive patients had much shorter PFS compared with PD-L1 negative patients using the established cutoff of 5%. A possible explanation is that the PD-L1 (+) CTCs reflect an immunosuppressive tumor microenvironment, which promotes tumor relapse. In addition, with the increased capacity of evading the immune system, PD-L1 (+) CTCs may have a higher metastatic potential.

In addition, the gene expression of PD-L1 in CTCs was found to be highly expressed in patients who had disease progression within 9 months compared to those who had stable disease for 9 months or more. The high expression of PD-L1 mRNA in our poor prognosis group also supports the prognostic value of PD-L1. The high purity of captured CTCs within the GO chip enabled downstream gene analysis such as qPCR. However, as it is unrealistic to achieve 100% purity, mRNA was extracted from a mixed population of CTCs and WBCs captured in the GO chip. Thus the mRNA levels detected here contained signals from both CTCs and the blood cells. This limitation could be overcome by developing single cell gene analysis technique to differentiate the CTC population with the contaminating blood cells.

The current study is limited due to the small patient cohort. Also the treatment plans and duration varied among patients. The impact of concurrent chemotherapy should be further explored. Undoubtedly, a significantly larger cohort can generate more reliable insights into the changes of PD-L1 expression under different treatment plans. Nevertheless, our data was the first to show that the transient increase of PD-L1 expression during radiation, which implied that PD-L1 expression should be monitored throughout treatment.

To create successful combinations of immunotherapy, radiation, and chemotherapy, many concerns need to be addressed such as how to select patients and how to choose the optimal treatment regimen and sequence with PD-1/PD-L1 inhibitors^12,13^. CTC enumeration and molecular characterization can be a valuable tool to help in this decision making process as CTCs can be sampled frequently without an invasive procedure. As we demonstrated the feasibility of monitoring PD-L1 (+) CTCs in this study, this CTC subgroup could potentially act as a biomarker to monitor the immune activation in tumor to facilitate treatment selection for combination with immunotherapies. Future efforts should incorporate dynamic monitoring of PD-L1(+) CTCs for the timed administration of radiation and PD-1/PD-L1 inhibitors in NSCLC patients.

## Methods

### GO chip production and surface functionalization

The GO Chip consists of a microfluidic chamber and a substrate coated with graphene oxide (GO) nanosheets where the antibodies are tethered (Fig. 1). Chips were fabricated and functionalized as described previously^27^. In brief, Graphene oxide nanosheets were adsorbed onto the silicon surface patterned with gold. As GO were functionalized by phospholipid–polyethylene–glyco-amine (PL–PEG–NH2), N-g-maleimidobutyryloxy succinimide ester (GMBS) was introduced, which has N-hydroxysuccinimide (NHS) esters that reacted with the amine groups of the graphene oxide– PEG to form amide bonds. Subsequently, the sulfhydrylgroup on NeutrAvidin reacted with GMBS and then biotinylated antibody for cell capture bound with NeturiAvidin. The PDMS top layer was fabricated using standard soft lithography. Master molds were fabricated using SU8-2025 photoresist (Microchem Corp.). A 10:1 ratio of polydimethylsiloxane (PDMS) polymer to curing agent (Dow-Corning) was well mixed and de-bubbled prior to pouring over the SU8 molds. After curing at 65 °C for over 6 hours, PDMS were manually cut from the mold, trimmed to size, and inlet/outlet holes were punched. Cr and Au films were deposited onto a silicon oxide coated silicon wafer by evaporation and were patterned by conventional photolithography. Patterned silicon substrates were dipped in a GO suspension for 10 minutes and rinsed with DI water and isopropanol. Then a silicon substrate and a PDMS chamber were bonded by corona discharge treatment to form a microfluidic chamber. The device was infused with N-γ-maleimidobutyryl-oxysuccinimide ester (GMBS) in ethanol. After a 30-minute incubation, the excess GMBS solution was washed out by ethanol. NeutrAvidin in phosphate buffered saline (PBS, Gibco) was flowed through the chip and incubated for 50 minutes followed by PBS washing. A biotinylated antibody cocktail (anti-EpCAM, anti-CD133 and anti-EGFR at a concentration of 10 μg/ml) was flowed through the chip and incubated for 30 minutes. After washing with PBS, 3% bovine serum albumin (BSA, Sigma-Aldrich) blocking solution was injected and incubated for 30 minutes.

### Cell preparation

Cell culture reagents were purchased from ThermoFisher Scientific unless otherwise specified. H441 (provided by David Beer lab) and H1650 cells (ATCC) were cultured in RPMI medium containing 10% fetal bovine serum and 1% penicillin–streptomycin solution. When cells reached 70–80% confluence, they were collected. To perform the capture efficiency experiments, cells were labeled with a green cell tracking dye (Invitrogen, CellTracker Green CMFDA, C7025).

### Human blood sample collection and processing

Blood samples were drawn from NSCLC patients and healthy donors after obtaining informed consent under University of Michigan and Ann Arbor Veterans Affairs Hospital Institutional Review Board-approved protocols. To be eligible for the study, patients had to have non-small cell lung cancer that was unresectable due to stage or medical reasons. Only patients planning to undergo radiation therapy or chemoradiation therapy were eligible. Samples were obtained at baseline (prior to radiation, visit 1), during radiation therapy (after 3 weeks for a fractionated course or the day of the final treatment for patients receiving stereotactic body radiotherapy, visit 2), and at a subsequent follow up visit (visit 3). Radiation therapy and chemotherapy were administered per standard of care. Patient samples were coded an anonymized at the time of collection. All samples were collected in EDTA tubes and were processed within 3 hours. All methods were carried out in accordance with relevant guidelines. 1 ml blood was flowed through each device at 1 ml/hr by a syringe pump. The captured cells were then washed with PBS, fixed with 4% paraformaldehyde (PFA), and stored at 4 °C until immunofluorescent staining.

### Immunofluorescence staining of isolated CTCs

Cells were permeabilized with 0.2% Triton-X (Sigma-Aldrich) and incubated for 30 min followed by a PBS wash. The device was incubated for 30 min with blocking buffer containing 2% normal goat serum and 3% BSA. Anti-cytokeratin (Pan) (mouse IgG1, MCA1907, Bio-Rad), anti-CD45 (mouse IgG2a, MCA87GA, Bio-Rad) and anti-PD-L1 (mouse IgG2b, 329702, BioLegend) antibodies were flowed through the graphene oxide chip, incubated for 1 hour, and washed with PBS. Anti-cytokeratin (Pan), anti-CD45 and anti-PD-L1 were probed respectively with Alexa Fluor 546 mouse IgG1 (A-21123, Invitrogen), Alexa Fluor 488 mouse IgG2a (A-21131, Invitrogen), and Alexa Fluor 647 mouse IgG2b (A-21242, Invitrogen). The secondary antibodies were flowed through the graphene oxide chip, incubated for 1 hour, and washed with PBS. To stain the nuclei of the captured cells, DAPI (4′,6-Diamidino-2-Phenylindole, Dihydrochloride) (Invitrogen) was flowed through the device. The device was incubated for 15 min and washed with PBS. The device was imaged and analyzed using Nikon Eclipse Ti fluorescence microscope and NIS-Elements software. All chips were imaged and analyzed within a week to ensure that there is no adverse effect to the putative CTCs. The PD-L1 expression is measured by Nikon NIS-Elements software using the area of the entire cells. The average pixel intensity of each cell was subtracted from the average pixel intensity of the local background for each image. The threshold for PD-L1 positivity is set based on the 95th percentile of the intensity observed in the negative controls (PD-L1 negative cell line and healthy control samples) (Table S6).

### RNA extraction and RT-qPCR

Arcturus PicoPure RNA Extraction buffer (Life Technologies) was flowed through the chip to lyse the captured cells immediately after PBS wash. After incubation at 42 °C for 30 min, the device was washed with water and the effluent were collected. The effluents, which were the total RNA samples, were stored at −80 °C until cDNA preparation. The cDNAs was synthesized using Ambion kit (Life Technologies) from total RNA samples and then pre-amplified using the TaqMan gene expression assays (Life Technologies) for PD-L1 (Hs01125301_m1) and housekeeping genes GAPDH (Hs02758991_g1), ACTB (Hs01060665_g1), and UBB (Hs04401230_gH). The expression patterns of preamplified cDNAs were analyzed by a quantitative polymerase chain reaction (qPCR) using the same TaqMan gene expression assays and the BioMark HD system (Fluidigm). We used no template control and also healthy control blood sample as controls for the gene expression experiments. Ct values were converted to Log2Exp values using R script package and shown in figures such as heatmap clustering.

### Statistical analysis

The CTC counts between patient samples and healthy control samples, the PD-L1 (+) CTC counts and the proportion of PD-L1 (+) CTCs between different visits were compared using the Wilcoxon signed-rank test. To analyze the expression levels of PD-L1 between different patient groups, each transcript was normalized to the average of three house-keeping genes (HKGs: GAPDH, ACTB, and UBB), and reported as −ΔC_T_, where ΔC_T_ = C_T gene_ − C_T (HKGs)_. The Ct values equal or above 35 were considered as no expression (29). Repeated measures ANOVA models were used to compare −ΔC_T_ between patient groups. The model includes group (poor/good prognosis), time points (before, during and after) and interaction between group and time points. Furthermore, a random effect is included in the model to consider the correlation between measurements within the same subject. Progression-free survival was defined as the time from diagnosis to progression or death, whichever occurs first. Patients who were alive and free-of-progression were censored at the time of last follow up. Progression was based off RECIST version 1.1. The Kaplan-Meier method was used to estimate the survival functions and Log-rank test was used for comparing survival functions for the dichotomized CTCs and PD-L1. Significance is determined if p < 0.05. All analyses were conducted using SAS (version 9.4, SAS Institute, Cary, NC).

## Electronic supplementary material

Supplementary Material

## Data Availability

The datasets generated during and/or analyzed during the current study are available from the corresponding author on reasonable request.
