# Supplementary material for: PD-L1 Expression in Circulating Tumor Cells Increases during Radio(chemo)therapy and Indicates Poor Prognosis in Non-small Cell Lung Cancer
Source: Sci Rep. 2019 Jan 24;9:566. doi: 10.1038/s41598-018-36096-7 (PMC6345864; doi:10.1038/s41598-018-36096-7)
Supplement: Supplementary file 1 — Supplementary Material [file 41598_2018_36096_MOESM1_ESM.pdf]

Supplementary Material

PD-L1 Expression in Circulating Tumor Cells Increases during Radio(chemo)therapy and Indicates Poor Prognosis in Non-small Cell Lung Cancer

Yang Wang[1,2], Tae Hyun Kim[2,3], Shamileh Fouladdel[2,4], Zhuo Zhang[1,2], Payal Soni[5], Angel Qin[4], Lili Zhao[6], Ebrahim Azizi[2,4], Theodore S. Lawrence[5], Nithya Ramnath[4], Kyle C. Cuneo [5,7]*, Sunitha Nagrath[1,2,8]*

[1]Department of Chemical Engineering, University of Michigan, Ann Arbor, MI
[2]Biointerfaces Institute, University of Michigan, Ann Arbor, MI
[3]Department of Electrical Engineering and Computer Science, University of Michigan, Ann Arbor, MI, USA
[4]Department of Internal Medicine, University of Michigan Comprehensive Cancer Center, Ann Arbor, MI
[5]Department of Radiation Oncology, University of Michigan Medical School, Ann Arbor, MI, USA
[6]Department of Biostatistics, University of Michigan, Ann Arbor, MI
[7]Veterans Administration Ann Arbor Healthcare System, Ann Arbor, MI 48105
[8]Translational Oncology Program, University of Michigan Health System, Ann Arbor, MI.

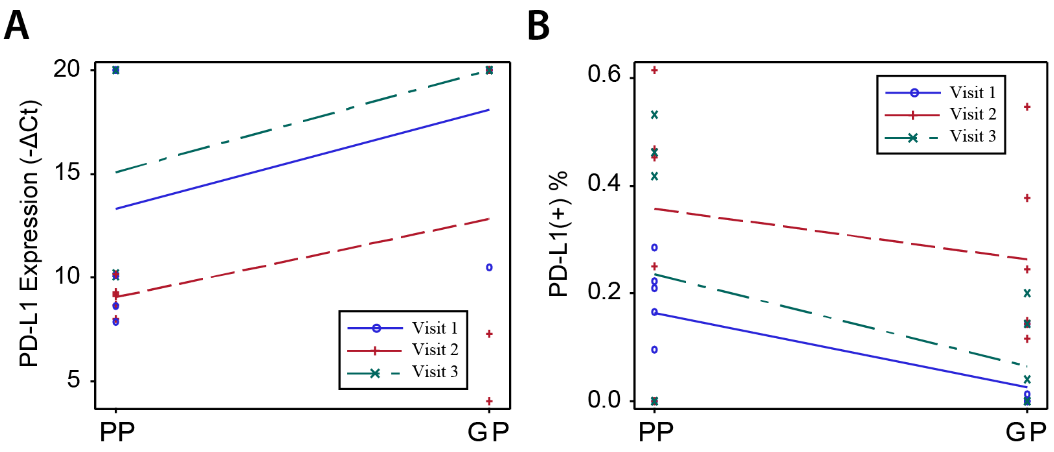

Figure S1. (A) The interaction plot of mRNA expression level (-ΔC_t) of PD-L1 (A) in PP samples (n=15) versus GP samples (n=14). (B) The interaction plot of PD-L1 (+) CTCs % in PP samples (n=17) versus GP samples (n=18). PP stands for poor prognosis and GP stands for good prognosis.

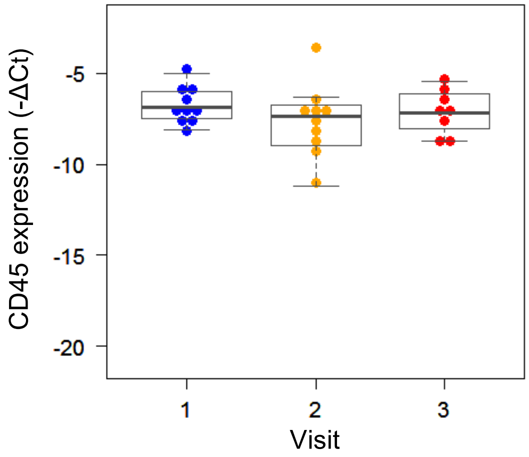

Figure S2. mRNA level (-ΔC_t) of CD45 in visit 1 samples (n=11), visit 2 samples (n=11) and visit 3 samples (n=9).

Table S1. Demographics of patients.

Patient	Age	Sex	Histology	T	N	M	RT dose	RT fraction size	Concur-rent chemo	Concurrent chemo drugs	Adjuv-ant chemo
Patient 1	60	Male	Adenocarcin-oma	2a	1	0	60	2	No	N/A	No
Patient 2	79	Male	Carcinoma	2	0	0	70	2	No	N/A	No
Patient 3	64	Male	Adenocarcin-oma	1	0	0	50	10	No	N/A	No
Patient 4	73	Male	Squamous	1a	3	0	50	5	No	N/A	No
Patient 5	72	Male	Squamous	2a	1	0	60	2	Yes	Carboplatin/Taxol	No
Patient 6	67	Male	Sarcomatoid carcinoma	3	2	0	60	2	Yes	Carboplatin/Taxol	No
Patient 7	65	Male	Squamous	4	3	0	60	2	Yes	Carboplatin/Taxol	No
Patient 8	63	Male	Squamous	2b	0	0	60	2	Yes	Carboplatin/Taxol	No
Patient 9	82	Male	Squamous	3	2	0	60	2	Yes	Carboplatin/Taxol	No
Patient 10	60	Male	Adenocarcin-oma	4	3	0	60	2	Yes	Carboplatin/Taxol	Yes
Patient 11	57	Male	Squamous	1b	3	0	60	2	Yes	Carboplatin/Taxol	No
Patient 12	67	Male	Squamous	2b	2	0	60	2	Yes	Cisplatin/Etoposide	No
Patient 13	66	Male	Squamous	2	0	0	50	5	No	N/A	No

Table S2. The number of CK (+)/CD45 (-) cells in 6 healthy control samples.

Healthy Control	Age	Gender	PD-L1(+)/CK(+) /CD45(-) cells (/ml)	PD-L1(-)/CK(+) /CD45(-) cells (/ml)	Total CK+/CD45- cells (/ml)
HC1	89	Male	0	2	2
HC2	27	Female	0	2	2
HC3	27	Female	1	2	3
HC4	28	Female	0	2	2
HC5	20	Male	1	1	2
HC6	32	Male	0	3	3

Table S3. A list of total CTC number, PD-L1 (+) CTC number and the fraction of PD-L1 (+) CTCs at different visits for 13 patients. ("N/A" stands for "not available".)

Patient	Visit 1			Visit 2			Visit 3		
	CTCs (/ml)	PD-L1 (+) CTCs (/ml)	Proportion of PD-L1 (+) CTCs	CTCs (/ml)	PD-L1 (+) CTCs (/ml)	Proportion of PD-L1 (+) CTCs	CTCs (/ml)	PD-L1 (+) CTCs (/ml)	Proportion of PD-L1 (+) CTCs
Patient 1	6	1	17%	70	43	61%	32	17	53%
Patient 2	48	0	0%	15.5	0.5	3%	17	2	12%
Patient 3	7	1	14%	5.5	3	55%	7	0	0%
Patient 4	5	0	0%	21	3	14%	9	0	0%
Patient 5	71	1	1%	57	21.5	38%	24	1	4%
Patient 6	14	0	0%	13	1.5	12%	4	0	0%
Patient 7	72	16	22%	31	14	45%	13	6	46%
Patient 8	62	13	21%	4	0	0%	12	5	42%
Patient 9	8	0	0%	22.5	5.5	24%	21	3	14%
Patient 10	17	0	0%	10	1.5	15%	5	1	20%
Patient 11	21	2	10%	6	1.5	25%	20	0	0%
Patient 12	4	0	0%	7.5	3.5	47%	9	0	0%
Patient 13	7	2	29%	N/A	N/A	N/A	33	0	0

Table S4. Statistics of total CTC number and PD-L1 (+) CTC % at different visits for 12 patients (Patient 1-12). (SD: standard deviation)

	CTC range (/ml)	Median of CTC number (/ml)	Average of CTC number (/ml)	SD of CTC number	PD-L1 (+) CTC % range	Median of PD-L1 (+) CTC %	Average of PD-L1 (+) CTC %	SD for PD-L1 (+) CTC %
Visit 1	5 - 72	15.5	27.9	26.0	0 - 22.2%	0.7%	7.1%	8.7%
Visit 2	4 - 70	14.3	21.9	20.3	0 - 61.4%	24.7%	28.3%	19.6%
Visit 3	4 - 24	12.5	14.4	8.2	0 - 53.1%	8.0%	15.9%	19.2%

Table S5. Progression-free survival (PFS) of patients. ("NED" stands for "no evidence of disease", censor = 0 for event and censor = 1 for censored records.)

Patient	Status	PFS (months)	PFS censor
Patient 1	Alive, Progressed	6.7	0
Patient 2	Alive, NED	2.3	1
Patient 3	Alive, NED	9.9	1
Patient 4	Alive, NED	9.6	1
Patient 5	Alive, NED	14.6	1
Patient 6	Alive, NED	18.9	1
Patient 7	Dead	6.5	0
Patient 8	Dead	5.7	0
Patient 9	Alive, NED	14.9	1
Patient 10	Alive, NED	17.5	1
Patient 11	Dead	7.4	0
Patient 12	Alive, Progressed	7.5	0
Patient 13	Alive, Progressed	8.3	0

Table S6. PD-L1 pixel intensities of negative control samples and selected PD-L1 (+) CTCs in patient samples.

Samples	PD-L1 pixel intensity difference	95% of PD-L1 pixel intensity difference
Healthy Control	0 - 24	21
PD-L1 negative cell line H1650	0 – 17	8.2
CTC 1	70	N/A
CTC 2	39	N/A
CTC 3	105	N/A
CTC 4	205	N/A
